# Is maternal trait anxiety a risk factor for late preterm and early term deliveries?

**DOI:** 10.1186/s12884-016-1070-1

**Published:** 2016-09-29

**Authors:** Margarete Erika Vollrath, Verena Sengpiel, Markus A. Landolt, Bo Jacobsson, Beatrice Latal

**Affiliations:** 1 Domain of Mental and Physical Health, Norwegian Institute of Public Health, Oslo, Norway; 2 Psychological Institute, University of Oslo, Oslo, Norway; 3 Department of Obstetrics and Gynecology, Sahlgrenska University Hospital, Gothenburg, Sweden; 4 University Children’s Hospital Zurich, Zurich, Switzerland; 5 Department of Child and Adolescent Health Psychology, Institute of Psychology, University of Zurich, Zurich, Switzerland; 6 Department of Obstetrics and Gynaecology, Sahlgrenska Academy, Gothenburg University, Gothenburg, Sweden; 7 Department of Genes and Environment, Norwegian Institute of Public Health, Oslo, Norway; 8 Child Development Center, University Children’s Hospital Zurich, Zurich, Switzerland

**Keywords:** Anxiety, Mental health, Personality, Pregnancy, Preterm, Prospective, Longitudinal, Women

## Abstract

**Background:**

Anxiety is associated with preterm deliveries in general (before week 37 of pregnancy), but is that also true for late preterm (weeks 34/0–36/6) and early term deliveries (weeks 37/0–38/6)? We aim to examine this association separately for spontaneous and provider-initiated deliveries.

**Methods:**

Participants were pregnant women from the Norwegian Mother and Child Cohort Study (MoBa), which has been following 95 200 pregnant women since 1999. After excluding pregnancies with serious health complications, 81 244 participants remained. National ultrasound records were used to delineate late preterm, early term, and full-term deliveries, which then were subdivided into spontaneous and provider-initiated deliveries. We measured trait anxiety based on two ratings of the anxiety items on the Symptom Checklist-8 (Acta Psychiatr Scand 87:364–7, 1993). Trait anxiety was transformed into categorizing the score at the mean and at ± 2 standard deviations.

**Results:**

Trait anxiety was substantially associated with late preterm and early term deliveries after adjusting for confounders. In the whole sample, women with the highest anxiety scores (+2 standard deviations) were more likely [(odds ratio (OR) = 1.7; 95 % confidence-interval (CI) 1.3-2.0)] to delivering *late preterm* than women with the lowest anxiety scores. Their odds of delivering *early term* were also high (OR = 1.4; CI 1.3-1.6). Women with spontaneous deliveries and the highest anxiety scores had higher odds (OR = 1.4; CI 1.1-1.8) of delivering late preterm and early term (OR = 1.3; CI = 1.3-1.5). The corresponding odds for women with provider-initiated deliveries were OR = 1.7 (CI = 1.2-2.4) for late preterm and OR = 1.3 for early term (CI = 1.01-1.6). Irrespective of delivery onset, women with provider-initiated deliveries had higher levels of anxiety than women delivering spontaneously. However, women with high anxiety were equally likely to have provider-initiated or spontaneous deliveries.

**Conclusions:**

This study is the first to show substantial associations between high levels of trait anxiety and late preterm delivery. Increased attention should be given to the mechanism underlying this association, including factors preceding the pregnancy. In addition, acute treatment should be offered to women displaying high levels of anxiety throughout pregnancy to avoid suffering for the mother and the child.

## Background

Research on preterm deliveries has been devoted to deliveries occurring before week 37 of gestation, with a focus on the earliest births. However, very early deliveries remain relatively rare. The substantial increase in shortened gestations noted in the last decades occurred late in the preterm period, between 34 and 36 weeks (*late preterm*), and in deliveries in weeks 37 and 38 (*early term*). These two forms of delivery onset together reach a share of up to 30 % of births in the United States [1, 2]. Not only newborns delivered late preterm but also newborns delivered early term have a higher risk for neonatal morbidity and later neurodevelopmental and behavioral problems [3, 4], as confirmed also recently in the Norwegian Mother and Child Cohort Study (MoBa) study [5, 6].

Therefore, there has been a continuous quest to find modifiable risk factors that could be treated or prevented to reduce the number of preterm deliveries. An important risk factor for spontaneous preterm deliveries in general is anxiety [7, 8]. Recently, there has also been a focus on birth anxiety, which involves fears concerning pregnancy and birth. However, we find this approach too narrow. Women experiencing fear of childbirth often have a history of anxiety disorders [8]. A way to approximate this history is to assess trait anxiety, which is a disposition to feel anxious, excessively worried, and nervous [9]. Trait anxiety lies on a continuum with anxiety disorders and increases the individual’s risk of feeling fearful and stressed in both harmless and harmful conditions. Feeling anxious and worried in turn triggers the biological stress response, which again activates neuroendocrinological mechanisms involving the hypothalamic-pituitary-adrenal axis [10].

Anxiety also increases the propensity to use psychoactive substances such as alcohol and tobacco, both before and during pregnancy, which again increases the risk for preterm births [11–13]. Moreover, shared genes for anxiety and addiction have been discovered [14]. Beyond the physiologically mediated risks, women experiencing anxiety may seek increased medical attention by requesting additional diagnostics or even demanding cesarean sections that are not medically indicated. Also, in a life course perspective, anxiety during pregnancy is probably an extension of dispositional anxiety before the pregnancy, which in turn may result in poorer reproductive health even before the first pregnancy [10, 15, 16].

This study addresses the association of trait anxiety with late preterm and early term delivery, both in the population of all pregnant women and in the subgroups of spontaneous and provider-initiated deliveries. We hypothesize that trait anxiety is associated with both gestational length, and that the association is higher in provider-oriented deliveries.

## Methods

### Study population

The Norwegian Institute of Public Health conducts a nationwide study of pregnant women, the Norwegian Mother and Child Cohort Study (MoBa). The study included 95 200 mothers recruited between 1999 and 2008 at about 100 hospitals and birth clinics across Norway, who were scheduled for a routine ultrasound examination [17, 18]. Among these, 40.6 % consented to participate [11] and completed questionnaires about their health and lifestyle at pregnancy weeks 17 and 30, and at 6, 18, and 36 months after childbirth. The MoBa study is linked to pregnancy and birth records in the Medical Birth Registry of Norway.

### Sample inclusion and exclusion criteria

MoBa releases updates every year; this study used the complete quality-assured MoBa dataset made available for research in 2013 (version 7). We included women who conceived spontaneously, gave birth to a singleton live-born infant, had a pre-pregnancy body mass index (BMI) from 14 to 50 [19], and provided valid information on anxiety on the week 17 and week 30 questionnaires. This reduced the sample to 90,083 women. We excluded women with deliveries before week 34/0 days and after week 40/6 days, women who participated with a second or third pregnancy in MoBa, and women with serious pre-pregnancy disorders (rheumatoid arthritis, kidney disease, chronic hypertension, heart disease, epilepsy, and diabetes type 1 or type 2). We also excluded women with infants who had Apgar scores less than 7 or had serious malformations. After we applied all the inclusion and exclusion criteria, 81,244 pregnant women remained in the sample.

### Measures

#### Preterm delivery

The length of all pregnancies in Norway is determined by second trimester ultrasound examination and recorded in days [20, 21]. If ultrasound information is missing, which applied to 1.7 % of deliveries in this study, pregnancy length was determined according to the date of the last menstruation. We distinguished three groups of gestational length in accordance with the recent criteria of the American College of Obstetrics and Gynecology [2]: *late preterm deliveries* (34/0–36/6 weeks), *early term deliveries* (37/0–38/9 weeks), and *full term deliveries* (39/0–40/6 weeks). Further, we subdivided the gestational age groups into spontaneous deliveries (delivery starting by spontaneous labor or spontaneous rupture of the membranes) and provider-initiated deliveries (induced labor or primary cesarean section) [20, 21]. All information was available from the Medical Birth Registry of Norway [18].

#### Trait anxiety

We used anxiety items completed on both questionnaires during pregnancy. We derived the items from a scale that was included in all MoBa questionnaires, the eight-item Symptom Checklist (SCL-8). The SCL-8 was specifically developed for medical patients and validated in seven European countries including Norway [22]. To avoid confounding with somatic symptoms, it only records emotional symptoms of anxiety and depression (four items for each construct). The four anxiety questions ask respondents about: (1) ‘feeling fearful’, (2) ‘feeling nervousness or shakiness inside’, (3) ‘worrying too much about things’, and (4) ‘feeling sudden fear for no reason.’ The response categories ranged from ‘not at all bothered’ (1) to ‘very bothered’ (4). We pooled the anxiety items into one average score for both time points, which had an excellent reliability (Cronbach’s alpha = 0.84) and high longitudinal stability (*r* = 0.83) with the identically constructed anxiety score measured at child age 3 years. We divided the pooled anxiety scale into four categories by means of cut-off points at the mean and ±2 standard deviations. The categories were: ‘very low’ = 1.0–1.25, ‘low to mean’ = 1.26–1.95, ‘mean to high’ =1.96–2.30, and ‘high’ = 2.31–4.00.

#### Confounders

Confounders are variables that are associated with both the exposure and the outcome. Therefore, we included only confounders that correlated with both gestational length and trait anxiety in preliminary analyses. We examined the variables maternal education, civil status, age, BMI before pregnancy, parity, daily smoking, alcohol consumption at least 1–3 times per month, urinary tract infections, gestational diabetes, and hypertension in pregnancy. We obtained information on most of these confounders from the Medical Birth Register of Norway, complemented by information from the questionnaires.

#### Data analyses

All analyses were carried out with IBM SPSS Statistics, version 22 [23] As recommended for longitudinal data, we imputed erroneous and missing values by means of maximum likelihood estimation, taking into account auxiliary, correlated data and information from later waves of the study [24]. For instance, missing maternal education was estimated by information on maternal age, income, and spouse’s/partner’s education and income. Missing data on smoking was imputed from information registered in the Medical Birth Register of Norway.

Second, we explored the associations of the confounders with gestational age group by means of univariate and multivariate multinomial regressions [23] separately for spontaneous and provider-initiated deliveries. In the two delivery onset groups, and the whole sample, neither gestational length nor anxiety was significantly associated with women’s alcohol use or urinary tract infections in bivariate associations. Hence, both variables were excluded. In multivariate analyses in the entire sample, marriage status, smoking, and planned pregnancy were not significantly associated with gestational length group. They were therefore excluded as well; education, parity, gestational diabetes, and hypertension remained in the multivariate analyses.

Third, to calculate associations of anxiety with gestational age group, we computed multinomial regression analyses with full term deliveries and ‘very low anxiety’ as reference. To determine whether anxiety and delivery onset interacted, we examined both main and interaction effects in the whole sample.

## Results

Women’s trait anxiety was distributed as follows in the whole sample: 2.3 % had very high anxiety, 3.3 % had high anxiety, 33.5 % had low anxiety, and 60.9 % had very low anxiety (numbers not shown in the table). The low frequencies in the high anxiety classes reflect that all traits tapping negative emotions show a reverse J-shaped form, with most individuals clustering on the ‘no negative emotions’ side.

Table 1 presents trait anxiety according to type of delivery (spontaneous vs. provider initiated) and gestation length within these categories. Data for gestation length is also given for the whole sample. In addition, the table shows demographic and pregnancy-related characteristics of the women according to delivery period and type. Looking at the ‘very high’ and ‘high’ anxiety categories, we find small proportions overall, ranging from 2.0 % to maximal 4.6 %. Women with provider-initiated deliveries have very high and high trait anxiety in all delivery periods more frequently than women with spontaneous deliveries (*Chi*^2^  =  202 615; *p*  ≤  0.000, *phi*   =  0.05). For example, 4.6 % of women delivering late preterm by provider-initiation report high trait anxiety, versus 2.8 % in the corresponding spontaneous group. When we collapse the two highest anxiety classes, the percentage is 5.1 versus 7.0.

Further, Table 1 shows that women in all groups were on average from 30 to 32 years old, and only 37 % to 43 % showed educational levels below college. Women delivering late preterm were the most often primiparous; women delivering early term by provider-initiation were the least often primiparous. Of note, but to be expected, are the elevated levels of gestational diabetes and pregnancy-related hypertension in the group with provider-initiated deliveries.

**Table 1:** Characteristics of mothers with late preterm, early term and full term deliveries by delivery onset

	Spontaneous deliveries^a^			Provider-initiated deliveries			All deliveries		
Gestational length^b^	Late preterm	Early term	Full term	Late preterm	Early term	Full term	Late preterm	Early term	Full term
N	1812	9701	56 527	1 007	4535	7824	2819	14054	64351
Anxiety Groups^c^	%	%	%	%	%	%	%	%	%
Very high	2.8	2.6	2.0	4.6	3.6	2.9	3.4	2.9	2.1
High	3.5	3.1	3.1	3.4	4.1	4.1	3.4	3.4	3.2
Low	36.6	33.7	32.6	38.2	37.9	35.8	37.2	35.0	33.0
Very low	57.1	60.6	62.3	53.8	54.5	57.1	55.9	58.7	61.7
Age, y; mean ± SD	29.6±5.0	29.7± 3.6	29.9±4.5	30.4±5.0	31.1±4.8	30.8±4.8	29.9 ± 5.0	30.1±4.7	30.1±4.5
Body Mass Index; mean ±SD	24.0±4.4	23.6±4.1	23.7±4.0	24.7±4.9	25.0±4.9	25.1±4.8	24.2 ± 4.6	24.1±4.4	23.9±4.1
Education less than college	43.6	39.8	37.4	42.2	43.1	39.7	43.1	40.9	37.7
Primiparous	53.9	44.9	42.8	48.6	34.2	45.3	52.0	41.6	43.1
Gestational diabetes	0.9	0.8	0.4	1.7	2.9	2.5	1.2	1.5	0.7
Gestational hypertension	5.7	3.9	2.7	37.9	15.9	16.4	17.2	7.6	4.3
Cesarean section	4.9	6.6	9.2	55.0	60.4	41.9	25.6	23.2	9.4

^a^Spontaneous deliveries (SD) = delivery onset by spontaneous labor or spontaneous rupture of the membranes

^b^Late preterm: weeks 34-36; early term: weeks 37-38; full term: weeks 39-41

^c^Cut-points at mean and ± 2 standard deviations. ‘Very low’ = 1.0-1.25; ‘low to mean = 1.26-1.95; ‘mean to high’ = 1.96-2.30; ‘high’ =  2.31-4.00

Table 2 shows the multinomial regression findings among spontaneous deliveries, provider-initiated deliveries, and all deliveries. Beginning with the unadjusted analyses, the most noticeable finding is that women with very high anxiety had odds of delivering *late preterm* OR = 1.5 and OR = 1.7 preterm in the spontaneous and provider-initiated groups, respectively. They had an OR = 1.8 in the entire sample. The odds of delivering early term were lower but still significant, ranging from 1.3 to 1.4 across women with spontaneous deliveries, provider-initiated deliveries and all deliveries. Women with high anxiety, the next category had no higher odds to deliver late preterm or early term. Women with low anxiety (compared to the reference group very low anxiety), had higher odds to deliver late preterm (OR = 1.2) and early term (OR = 1.1), but this finding was only significant in the entire sample. The adjustment for confounders did not reduce the associations of very high anxiety with late preterm and early term delivery remarkably.

**Table 2:** Associations of trait anxiety with gestation length in women with sponteneous deliveries, provider-intitiated deliveries, and all deliveries

		Spontaneous deliveries^a^			Provider-initiated deliveries			All deliveries		
	Gestation length^b^	Late preterm	Early term	Full term (reference)	Late preterm	Early term	Full term (reference)	Late preterm	Early term	Full term (reference)
	N	1812	9701	56 527	1 007	4535	7824	2819	14054	64351
Unadjusted analyses		OR (95% CI)^c^	OR (95% CI)	OR (95% CI)	OR (95% CI)	OR (95% CI)	OR (95% CI)	OR (95% CI)	OR (95% CI)	OR (95% CI)
Anxiety groups^d^	Very high	1.5 (1.1-2.0)	1.3 (1.2-1.5)	-	1.7 (1.2-2.3)	1.3 (1.0-1.6)	-	1.8 (1.4-2.2)	1.4 (1.3-1.6)	-
	High	1.2 (0.9-1.6)	1.0 (0.9-1.2)	-	0.9 (0.6-1.2)	1.0 (0.9-1.3)	-	1,2 (1.0-1.5)	1.1 1.0-1.2)	-
	Low	1.2 (1.1-1.4)	1.1 (1.0-1.1)	-	1.1 (1.0-1.3)	1.1 (1.0-1.2)	-	1.2 (1.2-1.4)	1.1 (1.1-1.2)	-
	Very low (reference)	-	-	-	-	-	-	-	-	-
Adjusted analyses		OR (95% CI)	OR (95% CI)	OR (95% CI)	OR (95% CI)	OR (95% CI)	OR (95% CI)	OR (95% CI)	OR (95% CI)	OR (95% CI)
Anxiety groups	Very high	1.4 (1.0-1.8)	1.3 (1.1-1.5)	-	1.7 (1.2-2.4)	1.3 (1.0-1.6)	-	1.7 (1.3-2.0)	1.4 (1.3-1.6)	-
	High	1.1 (0.9-1.5)	1.0 (0.9-1.1)	-	0.9 (0.6-1.3)	1.0 (0.9-1.3)	-	1.1 (0.9-1-4)	1.1 (1.0-1.2)	-
	Low	1.2 (1.0-1.3)	1.0 (1.0-1.1)	-	1.1 (1.0-1.2)	1.1 (1.0-1.2)	-	1.2 (1.1-1.3)	1.1 (1.1-1.2)	-
	Very low (reference)	-	-	-	-	-	-	-	-	-

^a^Spontaneous deliveries = deliveries beginning by spontaneous labor or spontaneous rupture of the membranes

^b^Late preterm: weeks 34-36; early term: weeks 37-38; full term: weeks 39-41

^c^OR (95% CI): Odds Ratio and 95% Confidence Interval

^d^Cut-points at mean, and ± 2 standard deviations. ‘Very low’ = 1.0-1.25; ‘low to mean = 1.26-1.95; ‘mean to high’ = 1.96-2.30; ‘high’ = 2.31-4.00

Interaction analyses showed that the odds of delivering late preterm or early term for women with high anxiety were not greater in provider-initiated deliveries as compared to spontaneous deliveries (multinomial regression, interaction term anxiety*delivery onset; *Chi2* = 4.34, *df* = 6, *P*   =  0.59).

## Discussion

In this study, high levels of trait anxiety predicted both late preterm and early term deliveries. Effect sizes were considerable: Women with very high trait anxiety had 80 % higher odds of delivering late preterm and 40 % higher odds of delivering early term. These associations were the same for spontaneous and provider-initiated deliveries, although women with provider-initiated deliveries had higher trait anxiety than women with spontaneous deliveries. We consider these results important, because late preterm delivery and early term delivery are frequent in modern societies, affecting millions of women worldwide and posing considerable risks to the children [1, 3–7].

These results extend and strengthen previous research on the risks posed by high anxiety for preterm birth as demonstrated in recent meta-analyses [4, 7]. Different pathological pathways may explain the association between trait anxiety and preterm birth. The main focus in the literature has been the hypothesis that anxiety and depression during pregnancy lead to stress, which in turn activates the maternal hypothalamic-pituitary-adrenal (HPA) axis, triggering a cascade of endocrinological, immunological, and vascular reactions that may alter the fetal environment [10, 25].

However, in line with Wadhwa [10], we subscribe to the notion that negative consequences of anxiety on pregnancy outcomes must be understood from a life course perspective. Reproductive health may already have been reduced before the first pregnancy. Already in late adolescence and early adulthood, anxiety is associated with risky health behaviors and health conditions that negatively affect female reproduction outcome, such as smoking, drinking, risky sexual behaviors, unplanned pregnancies, and selective abortions [11–13, 26–28]. Further pathways may involve maladaptive reactions due to excessive worries, self-monitoring, and hypochondriac reactions, leading to frequent visits to antenatal care units, requests for extra diagnostic procedures or cesarean sections, and low compliance with antenatal health advice [29, 30]. We could not test these pathways in this study, but a previous study from the MoBa showed that anxiety and depression predicted higher rates of cesarean section—independent of gestation length [31].

The finding that women undergoing a provider-initiated delivery more often had high trait anxiety should alert obstetricians. Even if their anxiety may be a consequence of knowing that the pregnancy is riskier rather than a cause, these women need medical attention, and symptom relief should be attempted.

This study has limitations. The MoBa study is observational and thus precludes causal interpretations. Even randomized controlled trials cannot firmly confirm causal relations. The sample is biased, comprising more highly educated, older, married or cohabitating, non-smoking women compared to the total population of women delivering during the same decade in Norway. However, several studies in the MoBa examining the associations of important exposures with pregnancy outcomes showed findings similar to those in the entire Norwegian birthing population [17, 32]. Moreover, we did not have a standard scale for trait anxiety but had to make do with a short scale, which is a disadvantage that is typical with all multifocal, large epidemiological studies. The scale has been validated in Norway, however [33]. Also, we had no measures of anxiety levels during the life course of the women prior to pregnancy, but given the stability of personality dispositions, we trust that we captured a stable trait. Most importantly, however, we cannot exclude the possibility that unmeasured third factors may cause both higher trait anxiety and preterm delivery.

## Conclusions

In conclusion, this study documented an association of high maternal trait anxiety with late preterm and early term delivery in one of the largest current mother and child cohort studies. Whether this association is mediated by stress, or other mechanisms, or can be explained largely by third variables may be examined by genetically informative studies such as family or twin studies or by molecular genetic linkage studies [34, 35]. As for clinical implications, antenatal screening for anxiety—and depression—should be a part of antenatal health care. Many pregnant women may not be aware that their anxiety is not normal, particularly if they have been very anxious all their lives. Treatment decisions should then be taken by specialists. Even if treatment does not affect the risk of preterm birth [36, 37], treating the anxiety will provide a health benefit to the mothers that will also affect how she takes care of the child later on.

## Abbreviations

BMI: Body mass index
MoBa: Norwegian Mother and Child Cohort Study
SCL: Symptom checklist
SCL-8: Symptom checklist with 8 items
